# The key to immunotherapy: how to choose better therapeutic biomarkers for patients with non-small cell lung cancer

**DOI:** 10.1186/s40364-022-00355-7

**Published:** 2022-03-07

**Authors:** Yue Pan, Yucheng Fu, Yue Zeng, Xiaohan Liu, Yurong Peng, Chunhong Hu, Chao Deng, Zhenhua Qiu, Jian Zou, Yuxuan Liu, Fang Wu

**Affiliations:** 1 Department of Oncology, The Second Xiangya Hospital, Central South University, Changsha, 410011 Hunan China; 2 Hunan University of Chinese Medicine, Changsha, 410208 Hunan People’s Republic of China; 3 Xiangya School of Medicine, Central South University, Changsha, 410013 Hunan People’s Republic of China; 4 Hunan Cancer Mega-Data Intelligent Application and Engineering Research Centre, Hunan, China; 5 Hunan Key Laboratory of Tumor Models and Individualized Medicine, The Second Xiangya Hospital, Central South University, Changsha, 410011 Hunan China; 6 Hunan Key Laboratory of Early Diagnosis and Precision Therapy in Lung Cancer, The Second Xiangya Hospital, Central South University, Changsha, 410011 Hunan China

**Keywords:** NSCLC, Immune checkpoint inhibitors, Biomarkers, PD-L1

## Abstract

Immunotherapy has become the standard of care for non-small cell lung cancer (NSCLC), either in combination or monotherapy. However, there are still some patients who cannot benefit from it. Immunization strategies for NSCLC are based on the expression of PD-L1 on tumor cells and TMB, and although these indicators have a certain predictive effect, their predictive performance is not good. Therefore, clinicians must make adjustments to recognize markers. This is a review article that summarized immunotherapeutic biomarkers according to the “seed-soil-environment”, generalizes primary resistance to immunotherapy, and summarizes the integration of markers.

## Introduction

Immune checkpoint inhibitors (ICIs) have been shown to be an effective new strategy in the treatment of lung cancer, and their use has been established as the standard treatment for patients with locally advanced/metastatic non-small cell lung cancer (NSCLC), whether in monotherapy or combination therapy. Despite this, many patients do not benefit from ICIs. Even in patients who initially respond to ICIs, disease progression may still eventually occur. Thus, identifying predictive biomarkers may help select those patients who are most likely to benefit from ICIs. Although PD-L1 expression and tumor mutation burden (TMB) have been widely used as biomarkers, both are incomplete. Due to the complex interactions between tumor cells, tumor microenvironments, and host immunity, we must build a multidimensional immune map to integrate complementary predictive markers for individual immunotherapy. This is a review article that discuss potential predictive biomarkers for ICIs in patients with NSCLC and the difficulties encountered in using these biomarkers and optimizing their routine clinical use.

## Tumor-related biomarkers

### PD-L1

**Fig. 1:**
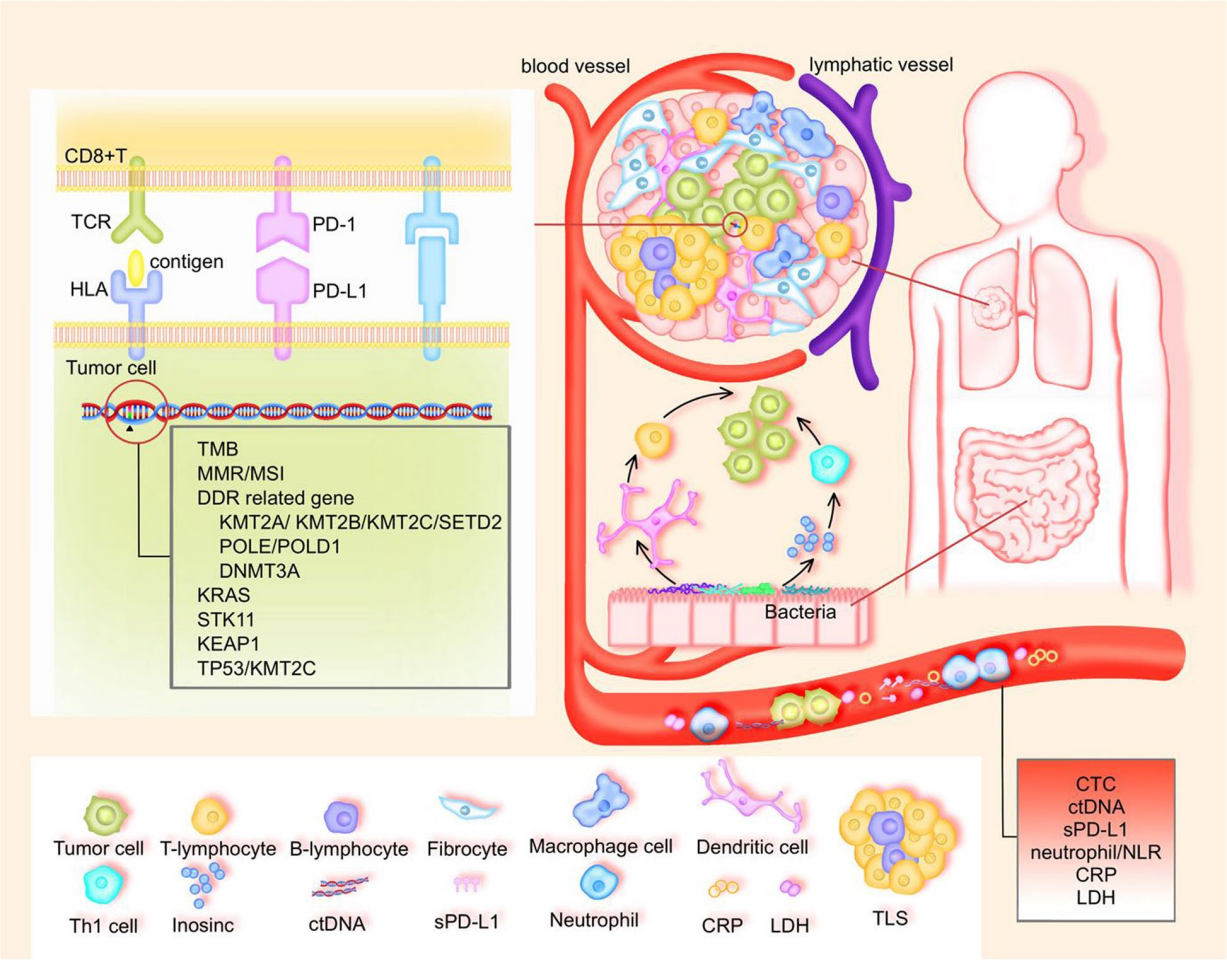
Potential biomarkers of the response to ICIs, according to “seed-soil-environment”

Currently, tumor PD-L1 expression is an approved biomarker to predict PD-(L)1 blockade in NSCLC (Fig. 1). Although PD-L1 expression is currently used to guide treatment decisions and regulatory approval, its expression may vary over time and across multiple tumor sites [1]. In the first-line treatment of patients with advanced NSCLC, tumor cell immunohistochemical PD-L1 expression is widely used to choose the patients who are most likely to benefit from ICIs. Furthermore, the KEYNOTE-024 and KEYNOTE-042 trials suggested that pembrolizumab could significantly improve the survival rate in patients with high expression levels of PD-L1 [2, 3]. However, not all trials have achieved a survival benefit with ICIs compared to standard platinum-based chemotherapy. For example, when compared to chemotherapy, Nivolumab was not associated with significantly longer progression-free survival (PFS) in previously untreated stage IV or relapsed NSCLC patients with 5% PD-L1 expression [4]. Moreover, Nivolumab plus ipilimumab in first-line NSCLC patients had longer overall survival (OS) compared to chemotherapy and was not associated with PD-L1 expression levels [5]. In addition, in EGFR/ALK-altered NSCLC, there was no difference in survival between patients with tumor PD-L1 < 1% vs. ≥ 1% [6]. Therefore, PD-L1 is not always reliable as a biomarker of immune efficacy in patients with advanced NSCLC.

ICIs can also be used as adjuvant or neoadjuvant therapy for early lung cancer, and PD-L1 does have a certain predictive effect on immunotherapy of early lung cancer. Multiple studies have shown that the expression of PD-L1 is related to the main pathological response (MPR) of adjuvant immunotherapy in early lung cancer [7–9]. Additionally, for the first time the extent of the pathological response to a neoadjuvant ICI has been found to be an independent prognostic factor of OS and disease-free survival (DFS) in NSCLC [10]. What’s more, in IB-IIIA NSCLC of PD-L1 TC > 1%, adjunctive treatment with atezolizumab reduced the risk of disease recurrence or death by 34%, and the greatest DFS benefit was observed in a PD-L1 TC > 50% population [11]. However, in the CheckMate816 trial, pathologic complete response rates showed a consistent benefit in patients with different PD-L1 expressions [12].

Although PD-L1 does have a certain predictive effect, the effect is still not perfect, possibly due to the following reasons. First, different detection methods for this index can give different results, which leads to “misclassification” of PD-L1 status for some patients [13]. Specifically, the manual evaluation of TPS by pathologists has practical limitations including intra/inter-observer bias, variation in subjectivity on areas of interest, and intensive labor requirements. Recently, the clinical performance of an AI-model in WSI-level tests has become comparable with assessment by pathologists. The AI model can accurately predict the tumor response and PFS of ICI treatment in advanced NSCLC [14].

In addition, PD-L1 is glycosylated at multiple sites, which prevents it from being recognized by PD-L1 detecting antibodies. Deglycosylation can make PD-L1 easier to detect, thus improving its predictive effect as a biomarker [15].

Second, there is frequently tumor heterogeneity in different regions of lesions, and the expression of PD-L1 in the tumor is a dynamic process. Third, previous treatment with radiotherapy in patients with advanced NSCLC typically results in longer PFS and OS with pembrolizumab treatment than those seen in patients who did not have previous radiotherapy [16]. This suggests that immune response can be related to the patient’s previous treatment, rather than just the expression of PD-L1.

### Tumor mutation burden

Tumor mutation burden (TMB) is another predictor for ICI efficacy (Fig. 1) and is calculated as the total number of nonsynonymous somatic mutations of the genomics coding area. These mutations can lead to neoantigen formation and contribute to the immunogenicity of the tumor [17, 18]. Indels in particular are a highly immunogenic mutational class, which can trigger an increased abundance of neoantigens and greater mutant-binding specificity [17]. This means that not only the number of mutations but the quality of mutations as well are decisive factors in the production of a new antigen. Additionally, high TMB is related to increased neoantigens and tumor infiltrating lymphocytes [19]; patients with high TMB, (TMB-H patients) have been found to have significantly longer PFS and OS in advanced NSCLC [20]. In one open-label, multipart, phase 3 trial, PFS was significantly longer with first-line nivolumab plus ipilimumab than with chemotherapy among patients with NSCLC, irrespective of PD-L1 expression levels [21].

In a study of early lung cancer, the CheckMate159 trial showed that mutation-associated neo-antigen (MANA) predicts the efficacy of neoadjuvant immunotherapy [22]. However, the NADIM study of neoadjuvant chemoimmunotherapy for resectable stage IIIA NSCLC showed that TMB failed to predict MPR, complete pathologic remission (CPR), and PFS [23].

TMB also has shortcomings as a marker of therapeutic effects. First, similar to PD-L1, the detection methods for TMB are still not uniform. Whole exome sequencing (WES) has traditionally been used to assess TMB, but high cost limits its widespread use. However, alternative methods such as next-generation sequencing (NGS) effectively stratify patients by likelihood of response [24]. Two NGS panels, the Oncomine Tumor Mutation Load Assay (OMLA) and the FoundationOne (F1), were compared by researchers to select the most accurate TMB prediction panel. They found that the TMB estimated by OMLA correlated more strongly with WES-derived TMB than TMB estimated by F1 [25]. In addition, TMB-H tumors, defined as the detection of ≥10 mutations/megabase (mut/Mb) in tumor cells using a tissue-based assay such as the FoundationOneCDx (F1CDx) assay, may be more likely to respond to some ICIs [25]. Although terms such as “low TMB” and “high TMB” are widely used, there are no clearly defined thresholds that define high or low levels [26].

Second, in recent years, intratumor heterogeneity (ITH) has become a hot topic [27]. In melanoma, compared with TMB, ITH has a greater impact on the efficacy of immunotherapy. The higher the ITH, the more easily the immune system’s ability to fight cancer is suppressed, so cancer progresses more quickly [28]. However, these current studies focus on clinical samples and mouse models of melanoma, and more data are needed to validate the findings. Finally, there is a knowledge gap regarding the definition of TMB, testing for TMB, and implementation of TMB status in clinical decision making [29]. Education on community oncology may be able to improve its application and adoption in solid tumors to improve the prognoses of patients.

### Damage response and repair

Damage response and repair (DRR) gene alterations in DNA are associated with increased tumor-infiltrating lymphocytes, higher genomic instability, and higher TMB in cancer [30, 31]. Mutations in the DDR gene are also associated with higher immunity [32], are common in NSCLC, and are associated with clinical outcomes in patients with NSCLC treated with ICI [30, 32]. Furthermore, DNA mismatch repair (MMR) is a method for identifying and repairing mutations that may occur during DNA replication and recombination. Specifically, systemic microsatellite instability (MSI) is a genetically hyper-mutational state that is a phenotypic result of MMR deficiency (dMMR). Although the prognostic significance of MMR and MSI-H have been established in colorectal and endometrial cancers, the role of MMR and MSI-H in lung cancer remains unclear. When using ICIs, patients with at least one deleterious mutation and another deleterious or indeterminate mutation generally have the best PFS or OS results, but do not respond to chemotherapy. This means the DDR pathway with harmful mutations may be a potential predictor for immunotherapy success [33].

Importantly, the overlap of TMB, MSI, and PD-L1 is different in different cancer types; only 0.6% of cases are positive for all three indicators [34]. In MMR-deficient cancers, most of the mutated neoantigens make them very sensitive to immune checkpoint blockade, regardless of the tissue origin of the cancer [35]. Therefore, some ICIs have been used to treat unresectable solid tumors with high MSI or dMMR [36, 37]. DNA repair gene mutations are related to tumor mutations and increased neoantigen load, which in turn is related to the greater infiltration of tumors by activated T cells, which leads to specific immunophenotypes in lung squamous cell carcinoma [38].

Previous studies have shown that many histone methyltransferases (HMTs) are recruited to DNA damage sites where they post-translationally modified chromatin to regulate chromatin-based DDR activities and that the loss of function (LOF) variants of HMTs may be related to genome instability and TMB [39]. Liang et al. found that 4 LOF variants of HMTs, KMT2A, KMT2B, KMT2C, and SETD2, are associated with better results of ICIs treatment [39]. In particular, both POLE and POLD1 encode the catalytic subunit of polymerase enzyme complexes involved in DNA replication and repair, and the association POLE/POLD1 mutations were associated with longer median OS in NSCLC (Undefined vs. 12.00 months, *P* = 0.05) [40]. In addition, among 600 NSCLC patients, deleterious mutations in the DNA methyltransferase 3A (DNMT3A) gene has been found to be the most significant alteration enriched in ICI responders versus nonresponders [41], and DNMT3A loss has been found to be associated with significantly higher ORR, (50% vs. 20.5%, *P* < 0.001), longer mPFS (9.2 vs. 2.9 months, HR 0.60, *P* < 0.01), and mOS (23.1 s 12.1 months, HR 0.59, *P* = 0.01) among DNMT3AMUT compared to DNMT3AWT NSCLCs [41].

### Tumor specific genes

Low-load TP53 mutations can predict PFS benefit in NSCLC with ICIs [42]. The reason may be that compared with the high TP53 variant allele fraction (VAF) group and the wild TP53 group, the low-load TP53 VAF group typically shows more immune cell infiltration and higher PD-L1 expression. Furthermore, the TP53/KMT2C common mutation can predict the response of NSCLC patients to ICI as well. Soecifically, one study found that PFS was significantly better in patients with co-mutation, possibly because patients with co-mutation of TP53/KMT2C had significantly higher initial CD8(+) T cells, initial B cells, Th1 cells, and Th2 cells in their tumor microenvironments (TME) [43].

Another type of mutation, KRAS mutations, are correlated with an inflammatory tumor microenvironment and tumor immunogenicity, resulting in good response to ICIs in advanced patients [44]. However, data on the impact of co-occurring STK11 mutations on outcomes are conflicting. Co-mutation of STK11 is associated with poor prognosis in KRAS G12C mutant lung adenocarcinoma patients treated with first-line ICIs [45]. In addition to co-mutations, a meta-analysis showed that mutations in STK11 and KEAP1 may adversely affect survival outcomes after ICIs in NSCLC patients [46]. Interestingly, KRAS mutations can predict PFS in NSCLC patients treated with dovalizumab after concurrent chemoradiotherapy, and mPFS was significantly shorter in patients with KRAS mutations than in patients without driver mutations [47]. This may be due to different patient stages in the studies and different immune microenvironments after radiotherapy.

Finally, a subset of patients suffer rapid tumor growth after ICIs, which is known as hyper-progressive disease (HPD). These HPD mutations have been found to be significantly associated with TMB levels and the occurrence of some driver genes, but are not correlated with PD-L1 expression [48]. Although the mechanism of HPD has not been fully elucidated, some genomic alterations, such as CDKN2A/CDKN2B loss and MDM2/MDM4 amplification have been reported to occur in tumors with ICI-related HPD. Therefore, timely detection of patients who may not benefit from immunotherapy in order to select other, more effective treatment options may improve outcomes for these patients.

## Tumor microenvironment

The tumor microenvironment (TME) is composed of immune cells, fibroblasts, blood vessels, and lymphatic tissues that surround tumor cells and has different abilities to induce favorable and unfavorable consequences of tumorigenesis [49, 50]. One study classified TME into four different types based on the status of tumor infiltrating lymphocytes (TILs) and PD-L1 expression [51] and found that the PFS and ORR of ICIs clearly differed according to the different TME types (ORR and median PFS; Type I: 64%, 14.5 months, Type II: 12%, 2.1 months, Type III: 24%, 3.6 months, Type IV: 41%, 10.8 months) [51]. More importantly, TME helps to identify patients with NSCLC who will benefit more from ICIs [52].

### T lymphocytes

Both CD8 + T cells and regulatory T (Treg) cells express PD-1, and the frequency of PD-1 + CD8+ T cells in the tumor microenvironment can predict the clinical efficacy of ICIs in a superior manner to other predictors, including PD-L1 expression and TMB [53]. The interaction between HLA/TCR is the first step in generating an immune response (Fig. 1). According to one study, in pembrolizumab monotherapy in patients with advanced NSCLC with high PD-L1 expression, the pre-treatment TCR diversity obtained from peripheral blood may be used as a good non-invasive biomarker of ORR, PFS, and toxicity, but it cannot be used as an biomarker of OS [54]. In addition, TCR repertoire measured in peripheral blood samples and tumor tissue may provide a useful tool for predicting the risk of recurrence after neoadjuvant durvalumab in patients with resectable stage IIIA(N2) NSCLC [55]. Furthermore, in patients with resectable NSCLC who underwent neoadjuvant therapy with atezolizumab, it has been shown that innate immune markers such as NK cells and NK-like T cells expressing ILT2- and NKG2A in the peripheral blood before treatment can be used to predict MPR [56].

Another emerging immune checkpoint molecule is T-cell immunoglobulin mucin-3 (TIM-3) [57]. Current studies have largely focused on T cells, found TIM-3 to be considered a marker of T cell depletion [58]. Antibodies that block TIM3 and PD-1 have a synergistic effect on tumor growth inhibition and improvement of tumor- antigen-specific CD8 + T cell response [59]. These results offer new hope for cancer patients unable to benefit from PD-1 antibodies. While we still have a lot to learn about TIM3, it may have good potential as a drug target for cancer.

In NSCLC, a meta-analysis that evaluated 7006 patients with stage I-IV NSCLC reported that CD8 + T cell infiltration was the best predictor of survival [60]. However, tumor-infiltrating immune cells are not a mature NSCLC biomarker because further data is needed to integrate and identify factors that affect the tumor microenvironment. Therefore, the incorporation of multiplex immunofluorescence may improve prediction of response and resistance to immunotherapy in NSCLC [61]. The clinical application of an artificial-intelligence-driven spatial TIL analyzer to predict the outcome of ICIs treatment of advanced NSCLC [62] has also recently been used in studies, but studies of individual TILs are always one-sided. Here, the inflammation score (IS) is defined as the proportion of all tumors in the WSI that contain 1 mm^2^ tiles that are classified as inflammatory immunophenotype (high TIL density within the cancer epithelium), and it is associated with good overall survival rates for multiple tumor types [62].

In addition to focusing on PFS and OS, if a patient has immune-related adverse events (irAEs), even if the ICI is effective, the drug causing the irAEs has to be discontinued. In this regard, baseline peripheral CD8 + T cells predict irAE occurrence and clinical outcome in patients with advanced NSCLC [63]. And HLA-I heterozygosity predicts the occurrence of irAE in NSCLC patients receiving ICIs [64]. In addition, differences in baseline gene expression may influence the incidence of irAE in chemotherapy-immunoneoadjuvant therapy in patients with resectable NSCLC [65].

### B lymphocytes

Existing tumor immunotherapy mainly focuses on killer T cells in the immune system. However, the B cells in the tertiary lymphoid structure (TLS) are related to the better treatment response of immunotherapy and provide a new tool for the prognosis prediction of tumor immunotherapy [66]. The TLS in patients with NSCLC characterized by clusters of mature dendritic cells (DCs) and T cells surrounded by B-cell follicles is shown in Fig. 1 [67, 68]. TLS, like TILs, are considered to be a predictor of increased survival, and B cell infiltration and TLS in soft tissue sarcomas are positively correlated with patient prognosis and response to ICIs. In one study, B cells were found to be the strongest prognostic factor, and patients in the B-cell-rich immune subgroup who received ICI during the clinical trial had the highest survival [69]. Similarly, TLS predicts lasting clinical benefit of ICIs in NSCLC PD-1-positive patients [70]. Although it remains to be studied which B cell subsets and TLSs promote the response to ICIs, TLSs provides new biological markers and guidance for the application and clinical decision making of ICIs.

### Dendritic cells

In the TME DC uptake, process, and present antigens and are all potent activators of the T-cell immune response [71], and PD-1/PD-L1 pathway inhibitors have brought revolutionary breakthroughs in clinical antitumor therapy. In fact, PD-L1 has two important ligands, PD-1 and B7.1 [72]. The B7.1 of DC binds to the CD28 receptor of T cells, a key step for T cells to gain functional activity, and the dendritic cells in the patient’s tumor from one study expressed both PD-L1 and B7.1. Here, excessive PD-L1 “buried” B7.1 in the cell membrane and prevented B7.1 from binding to CD28 on T cells, meaning that the antigen-presenting cells lost their ability to activate T cells. When patients use ICIs, a part of the antibody can bind to PD-L1 on DC, blocking the binding and embedding of PD-L1 and B7.1 so that B7.1 is released and can bind to CD28+ T again. The authors found that the high expression of DC-related genes in the tumor tissues of patients with kidney cancer and NSCLC before treatment is positively correlated with OS [72]. TIM3 is also expressed in the DC. In one study, after tim-3 knockout in DCs, the number of infiltrating CD8+ T cells increased, and some MHC class I antigen presentation genes were significantly elevated [73]. Therefore, it may be even more critical in boosting anti-tumor immunity. Several TIM3 antibodies are already in clinical trials, and we hope that these drugs will soon be available for the benefit of more patients.

### Macrophages

Macrophages are a double-edged sword in the TME (Fig. 1). Macrophages, as an important part of tumor stromal cells, can gather around blood vessels to induce angiogenesis and promote tumor invasion. However, they can also swallow cancer cells and reshape the TME itself. TAM cells are a type of tumor-associated immune cell that belongs to the macrophage cell line that infiltrate or are adjacent to tumor tissues, and there are also specific M2 morphological macrophages that exert immunosuppressive and tumor-promoting effects among them that are defined as TAM in a narrow sense. TAM cells can inhibit effector T cells through a variety of ways such as the escape and killing effect of tumor cells. To ensure the effect of ICIs, anti-PD-1 antibodies must be able to stay on the target points on the surface of T cells to prevent T cell failure. However, TAM can “rob” anti-PD-1 antibodies from the surface of CD8+ positive T cells, swallow them into the body, and make them ineffective [74]. Whether can be used as a predictor of the effectiveness of ICIs requires further experimentation. In a study of nearly 500 cases of NSCLC, the main immune cell type expressing PD-L1 was found to be CD68 macrophages. The level of PD-L1 in macrophages was significantly correlated with the level of PD-L1 in tumor cells and the infiltration of CD8 T cells, suggesting that high levels of PD-L1 are related to “hot” tumors. In immunotherapy patients, high levels of PD-L1 expression in macrophages are associated with longer OS [75, 76].

## Host-related biomarkers

### Peripheral blood

Traditionally, the markers that predict the efficacy of immunotherapy are mostly focused on tumors and TME; however, the host immune system plays a central role in driving the response to immunotherapy. Since biopsy of tissue samples is difficult to obtain, some alternative methods such as minimally invasive peripheral blood biopsy are becoming more and more popular. Peripheral blood contains DNA, RNA, and proteins released by tumor tissues, reflecting the dynamic changes in the TME (Fig. 1). The circulating tumor DNA (ctDNA) predicts OS in metastatic NSCLC [77], and a study of neoadjuvant ICIs combined with chemotherapy in the treatment of stage IIIA NSCLC showed the existence of a predictive effect of ctDNA levels on long-term survival before treatment [78]. What’s more, ctDNA quality-corrected bTMB can be used as a predictive biomarker [79, 80]. In the results of the Blood First Assay Screening Trial (BFAST) in patients with bTMB≥16, the ORR and OS of atezolizumab improved compared with chemotherapy [81]. In addition, combinatorial assessment of bTMB-tTMB impacts prediction of clinical outcome [82].

Cytological PD-L1 assessment also predicts ICIs response in NSCLC patients comparable to histology [83]. In recent years, a series of soluble immune checkpoints have been discovered in blood, for example soluble PD-1 (sPD-1) [84]. These soluble checkpoints are involved in positive or negative immune regulation, and changes in their plasma levels affect the development, prognosis, and treatment of cancer [85]. A meta-analysis reported that the levels of sPD-L1 in the blood of patients with various malignancies and high levels of sPD-L1 significantly predict the poor prognosis of patients with solid tumors [86].

Previously, neutrophils were deemed to be only inert bystanders, but current evidence suggests that tumors manipulate neutrophils, sometimes in the early stages of their differentiation process, to create different phenotypes and functional polarization states, which can change tumor behavior [87]. The immune cell composition of NSCLC is fundamentally different; here, neutrophils are the most common type of immune cells [88]. The OS of patients with baseline neutrophilia has been shown to decrease significantly, and neutropenia during treatment can attenuate its negative impact on prognoses in advanced NSCLC [89]. In addition, the neutrophil-lymphocyte ratio (NLR) can be used as a surrogate indicator to predict myeloid suppressor cell (MDSC) expansion after ICIs [90]. In patients treated with ICIs alone, high NLR is associated with poor survival in different PD-L1 expression categories, but chemotherapy combined with immunotherapy seems to overcome the poor survival caused by myeloid cell-mediated immunosuppression [90]. Moreover, low NLR may be a useful predictor of pseudoprogression in immunotherapy [91]. Therefore, reducing neutrophil may be one of the ways to improve the efficacy of ICIs in the future.

Even under aerobic conditions, tumor cells with strong metabolisms still need anaerobic glycolysis to obtain energy (known as the Warburg effect), and this process requires the up-regulation of most enzymes in the glycolytic pathway, including lactate dehydrogenase. High lactate dehydrogenase (LDH) is associated with an adverse prognosis in many solid tumors [92], and the same is true for immunotherapy patients [93]. Using the same principle of glycolysis of tumor cells, in patients receiving pembrolizumab monotherapy, PET-derived parameters (such as total glycolysis of the lesion and tumor metabolic volume) are related to the prognosis of the patients [94]. Systemic inflammation suggested by elevated CRP is a very strong indicator of poor prognosis for patients receiving immunity therapy and has potential negative predictive value [95]. Additionally, a prognostic nutritional index (PNI) containing CRP and NLR showed a trend towards being an independent prognostic factor for OS [96].

### Age

Although age-related immune dysfunction may cause differences in the efficacy of ICIs between young and elderly patients, the potential impact of age on the efficacy of ICIs is still unclear and controversial. One study showed that the TMB of the elderly group was higher than that of the younger group, and the OS was longer [97].

### Microbial factors

Two groundbreaking studies in 2013 showed that a complete gut microbiome is essential for the efficacy of anti-cancer therapy [98, 99], and in recent years, gut microbiota has become a hot topic (Fig. 1). Toxins associated with intestinal flora are associated with tumor formation and development [100]. The diversity of intestinal flora is closely related to the effectiveness of ICIs in patients with advanced NSCLC, and the higher the diversity of intestinal flora, the better the efficacy [101, 102]. Fecal microflora transplantation (FMT) from cancer patients that respond to ICIs into sterile or antibiotic-treated mice can improve the anti-tumor effect of ICIs, while transplantation of fecal microflora from nonresponsive patients does not [103]. Among these gut micro-organisms, *Akkermansia muciniphila* (Akk), has emerged as a potential hallmark of clinical benefit to ICI [104], and FMT combined with ICIs changes intestinal flora and improves tumor microenvironment to overcome ICIs resistance [105]. Future research should explore the predictive value of gut microbiota and broader interventions.

Antibiotic administration within 30 days prior to the commencement of tumor immunotherapy significantly shortens PFS and OS [106]. However, recent studies suggest that antibiotic use does not affect the efficacy of first-line chemotherapy combined with ICI in patients with advanced NSCLC [107]. Therefore, whether the use of antibiotics affects the antitumor efficacy of ICI still needs more research. In conclusion, although there is not enough data to show a causal relationship between specific bacteria, tumor species, ICI schemes, and efficacy, more attention should be paid to this in clinical diagnosis, treatment, and research, and the influence of antibiotics and other concomitant drugs should be taken into account as well.

### The integration of biomarkers

Immunotherapy involves antigen presentation, lymphocyte activation, as well as other aspects, so the use of one biomarker is always one-sided, and the combination of multiple indices has a more scientific predictive effect on immunotherapy. Additionally, the combined evaluation of multidimensional biomarkers has better efficacy prediction value [108]. There are also a growing number of clinical trials that combine immunotherapeutic markers (Table 1). For example, the combination of TMB and PD-L1 might maximize the predictive precision for patient stratification. A TMB 90th percentile was found to be correlated with longer PFS/OS to ICI among NSCLC with PD-L1 levels 50% and 1–49%, and longer PFS among those with PD-L1 < 1% [109]. Furthermore, the combination of CTL engagement with tumor cell and helper T lymphocytes along with increased expression of PD-L1, which represents enhanced endogenous immune reactivity, more accurately differentiates nonresponders to ICI compared to tumor cell PD-L1 alone and captures the importance of cellular interactions in the TME [110].

**Table 1:** Ongoing clinical trials to explore combined biomarkers of ICI in NSCLC

Identifier	Time Perspective	Patients	Treatment	Tumor-related biomarkers	TME	Host related biomarkers
NCT04918836	Prospective	Metastatic NSCLC	Initiation of ICI	–	lymphocyte immunophenotyping	autoantibodies, RF, LDH, complement (C3 C4), anti-tissue antibodies
NCT03578185	Retrospective	NSCLC	treated with ICI	Neoantigen, TMB, PD-1/PD-L1 expression	T-cell receptor and associated immune gene signature	fecal material
NCT04589013	Prospective	metastatic or locally advanced unresectable NSCLC	pembrolizumab + first line chemotherapy	PD-L1	CD8, FoxP3, PD1, CD163, CD15	–
NCT04629027	Retrospective	IIIB-IV non-squamous NSCLC	ICI	–	T cell marker	CTC
NCT04923802	Prospective	stage I-IV NSCLC	ICI	NGS-based genomic, transcriptomic, and methylomic profiling	–	blood samples
NCT04858828	Prospective	Advanced NSCLC	immunotherapy combined with chemotherapy in the first-line treatment	PD-L1,TMB, MSI, DDR,	TCR	blood samples
NCT04636047	Prospective	NSCLC	ICI	–	HLA,TCR	bTMB
NCT04804137	Prospective	Metastatic NSCLC	ICI	–	T cell sub populations, B lymphocytes	sputum, blood samples, intestinal microbiota
NCT04646837	Prospective	IB-IIIA NSCLC	neoadjuvant treatment	WES, GEP gene expression profiling, ctDNA	TCR	

In order to reflect the degree of immune cell invasion in the tumor microenvironment better, eight additional genes related to immune cell invasion and abundance in the TME were added to the interferon signaling pathway to form the tumor inflammation index (TIS) [111]. In the RATIONALE-307 study, TIS was found to predict the PFS benefit of tislelizumab combined with chemotherapy in advanced NSCLC [112], integrating the expression of PD-L1 in circulating leukocytes, platelets, and platelet particles and the expression biomarker of PD-L1 in tumor cells can better distinguish patients who may benefit from immunotherapy [113].

As clinicians, we can also conveniently score points based on the test results of peripheral blood combined with clinical features. The SIPS score has been combined with albumin and neutrophil counts to evaluate the prognostic effect of inflammatory biomarkers on first-line pembrolizumab therapy [114], and the predicted score based on NLR, age ≥ 65 years, female, never smoking, adenocarcinoma, can predict OS benefit of first-line pablizumab ± chemotherapy [115]. IN addition, a retrospective study of 62 patients with advanced NSCLC receiving front-line ICI was conducted to test the predictive effects of BMI and mEPSILoN score, where MEPSILoN was assessed by five clinical variables: smoking, ECOG PS, liver metastasis, LDH, and NLR. This score was associated with better OS benefits for ICI front-line treatment in patients with advanced NSCLC [116].

## Conclusions

The interaction between immune cells and tumor cells in the TME is complex, and combining multiple indicators to predict immune efficacy is the future trend based on recent research results. In addition, with the approval of a variety of ICIs and the availability of prices, clinicians can try to use ICIs for a period of time instead of predicting them in advance. Finally, future immune biomarkers should reveal the link of any immune block, and they should be used to help clarify when and how long to use ICIs. We belief that the coming era of immunotherapy will benefit more patients than in the past.

## Data Availability

Not applicable.

## Abbreviations

NSCLC: non-small cell lung cancer
ICIs: Immune checkpoint inhibitors
TMB: Tumor mutation burden
PFS: Progression-free survival
OS: Overall survival
MPR: Main pathological response
DFS: Disease-free survival
MANA: Mutation-associated neo-antigen
CPR: Complete pathologic remission
WES: Whole exome sequencing
NGS: Next generation sequencing
OMLA: Oncomine Tumor Mutation Load Assay
TIH: intratumor heterogeneity
DDR: Damage response and repair
MMR: Mismatch repair
MSI: Microsatellite instability
HMT: Histone methyltransferases
LOF: Loss of function
DNMT3A: DNA methyltransferase 3A
VAF: Variant allele fraction
TME: Tumor microenvironment
HPD: Hyper-progressive disease
TILs: tumor infiltrating lymphocytes
TIM-3: T-cell immunoglobulin mucin-3
IS: Inflammation Score
irAEs: immune-related adverse events
TLS: Tertiary lymphoid structure
DCs: Dendritic cells
ctDNA: Circulating tumor DNA
BFAST: Blood First Assay Screening Trial
sPD-1: soluble PD-1
NLR: Neutrophil-lymphocyte ratio
MDSC: Myeloid suppressor cell
LDH: Lactate dehydrogenase
PNI: Prognostic nutritional index
FMT: Fecal microflora transplantation
Akk: *Akkermansia muciniphila*
TIS: Tumor inflammation index
